# Harnessing national data systems to understand circumstances surrounding veteran suicide: linking Department of Veterans Affairs and National Violent Death Reporting System Data

**DOI:** 10.1186/s40621-024-00559-5

**Published:** 2025-01-21

**Authors:** Claire A. Hoffmire, Alexandra L. Schneider, Laurel A. Gaeddert, Joseph Logan, Julie A. Kittel, Ryan Holliday, Lindsey L. Monteith

**Affiliations:** 1 VA Rocky Mountain Mental Illness Research, Education and Clinical Center for Suicide Prevention, Aurora, CO USA; 2 Department of Physical Medicine and Rehabilitation, University of Colorado Anschutz Medical Campus, Aurora, CO USA; 3 Anderson College of Business and Computing, Regis University, Denver, CO USA; 4 Branch Development, Data & Insights, Edward Jones, Saint Louis, MO USA; 5 Division of HIV Prevention, Centers for Disease Control and Prevention, Atlanta, GA USA; 6 Department of Psychiatry, University of Colorado Anschutz Medical Campus, Aurora, CO USA

**Keywords:** Data linkage, Veteran, Suicide

## Abstract

**Background:**

Veterans are at elevated risk for suicide compared to non-Veteran U.S. adults. Data sources and analyses to inform prevention efforts, especially for those who do not use Department of Veterans Affairs (VA) healthcare services, are needed. This study aimed to link VA and CDC’s National Violent Death Reporting System (NVDRS) data to create a novel data source to characterize the circumstances precipitating and preceding suicide among Veterans, including among those who did not use VA healthcare.

**Methods:**

Multi-variable, multi-stage, deterministic linkage of VA-Department of Defense (DoD) Mortality Data Repository (MDR) and NVDRS-Restricted Access Database suicide and undetermined intent mortality records within 189 state-year strata (42 states, 2012–2018). Three linkage stages: (1) *exact* (matched on: age, sex, death date, underlying cause of death, day of month of birth, first initial of last name); (2) *probable* (all but one variable matched); (3) *possible* (all but 2 variables matched). Linkage success and accuracy of NVDRS-documented military history were assessed.

**Results:**

Across all state-years, 22,019 matches (89.20% of 24,685 MDR Veteran records) were identified (65.47% exact). When high missingness (2 + matching variables in > 10% of records; *n* = 23) or incomplete reporting (*n* = 12) state-years were excluded, match rate increased to 94.29% (77.15% exact). NVDRS-documented military history (ever served) was accurate for 87.79% of matched records, with an overall sensitivity of 84.62%. Sensitivity was lower for female (61.01%) and younger (17–39 years; 77.51%) Veterans.

**Conclusions:**

Accurate linkage of VA-DoD and NVDRS data is feasible and offers potential to improve understanding of circumstances surrounding suicide among Veterans.

## Introduction

In 2021, the Veteran suicide rate was 33.9 per 100,000, and the age- and sex-adjusted Veteran suicide rate rose 76.3% from 2001 to 2021 [1, 2]. Suicide risk among Veterans differs by sex, with a 2021 rate of 17.5 per 100.000 for females and 35.9 per 100,000 for males. Suicide rates and trends also differ for Veterans who did and did not use Veterans Health Administration (VHA) services in the year prior to death. Specifically, VHA users experienced a suicide rate of 41.5 per 100,000 in 2021, which was a 40.1% age- and sex-adjusted increase from 2001 while other Veterans (i.e., those who did not use VHA services in the year of or preceding death) experienced a lower 2021 suicide rate of 30.6 per 100,000 but a greater age- and sex-adjusted percent increase of 73.7% since 2001 [2]. These trends, coupled with the fact that the majority of Veterans do not use VHA services, means that most Veteran suicide deaths occur among those who have not recently used VHA services [1].

When evaluating suicide trends by sex, there is particular concern for female Veterans overall, and for both male and female Veterans not using VHA care. Specifically, among VHA Veterans, the age-adjusted suicide rate rose 24.5% for males and 87.1% for females, whereas among other Veterans, 62.6% and 93.7% increases were observed for males and females, respectively [2]. Accordingly, public health, community-based and gender-sensitive approaches to suicide prevention within the Department of Veterans Affairs’ (VA) are critical [1, 3].

Nonetheless, knowledge is lacking regarding why suicide rates are increasing more rapidly among non-VHA Veterans, and why this trend is particularly marked for females. One strategy to gain such knowledge is to improve the collection and integration of surveillance data on Veterans’ suicidal behavior [3]. The VA-Department of Defense Mortality Data Repository (MDR) and associated annual reports [4] have greatly advanced Veteran suicide surveillance by reporting rates and trends over time. However, there remains a need to expand the depth of Veteran suicide surveillance data, especially for those who did not use VHA services proximal to death. In particular, it is essential to increase what is known about suicide decedents prior to their deaths, which may help with identifying potential drivers of suicide risk and optimal prevention strategies. Descriptive, demographic information, such as that collected on death certificates, is important yet insufficient to meaningfully inform effective prevention strategies. More information on the preceding and precipitating circumstances of death, characteristics of the events, methods used, health-seeking behaviors, and personal relationships of decedents may help shed light on these deaths in a way that can better focus prevention efforts.

For Veterans who use VHA services, a wealth of clinical information is available from VHA medical records which can be analyzed to inform prevention efforts [5]. Additionally, some information on social determinants of health (SDOH) is available from VHA records for Veterans actively connected to and engaging in services (e.g., VA homeless programs [6] and justice-related services [7]). Conversely, clinical and SDOH information for the full Veteran population, particularly Veterans not using VHA services, is not readily available. Most examinations of non-VHA using Veterans rely upon surveys which, while valuable, often cannot feasibly reach sufficient sample sizes to study suicide mortality as an outcome, especially among minority Veteran subgroups, such as female Veterans.

National mortality data are another important source of information for investigating potential drivers of suicide risk in the broader Veteran population. In particular, the Centers for Disease Control and Prevention’s (CDC) National Violent Death Reporting System (NVDRS) has been used to study circumstance-based risk factors for suicide among those with a history of military service [7, 8]; however, the NVDRS military indicator does not specifically identify Veterans separated from service [9]. Prior to publication of the first annual VA Suicide Data Report in 2016, which reported on 2001–2014 mortality data [10], NVDRS data were used to estimate Veteran suicide rates relative to non-Veterans [11] and to derive indirect estimates comparing rates among Veterans with and without a history of VHA service use [12]. More recent analyses of NVDRS data have examined risk factors for suicide methods (e.g., firearms) among those with a history of military service [13] and have compared risk factors for suicide between those with and without a history of military service, overall [14] and by sex [15, 16]. Furthermore, at least two individual states, Colorado and Oregon, have linked their VDRS data with VA data to validate Veteran status and behavioral health variables, respectively [17, 18]. While findings from these studies are informative, concerns remain regarding the accuracy of the NVDRS military history indicator variable [19]. Furthermore, distinguishing between Veterans who do and do not use VHA services prior to death on a national level has not been possible with NVDRS data. As such, there remains a sizeable gap in knowledge to inform how best to curb rising suicide rates among Veterans not using VHA care.

To address this critical knowledge gap and answer a compelling “Call to Link Data to Answer Pressing Questions About Suicide Risk Among Veterans” [19], we aimed to demonstrate the feasibility of linking the NVDRS-Restricted Access Database (RAD) to VA-DoD MDR death records. The current manuscript describes the process and outcomes of a multi-variable, multi-stage, deterministic linkage approach. Secondarily, we aimed to: [1] measure the success and quality of this linkage process overall and across NVDRS state-years; and [2] assess the accuracy of the NVDRS military history variable, overall, and by sex and age. Achieving these aims is expected to support future analyses in support of the 2024 National Strategy for Suicide Prevention [20] and the VA’s National Strategy to provide gender-sensitive, community-based suicide prevention approaches for all Veterans [3].

## Methods

### Data sources

**Fig. 1:**
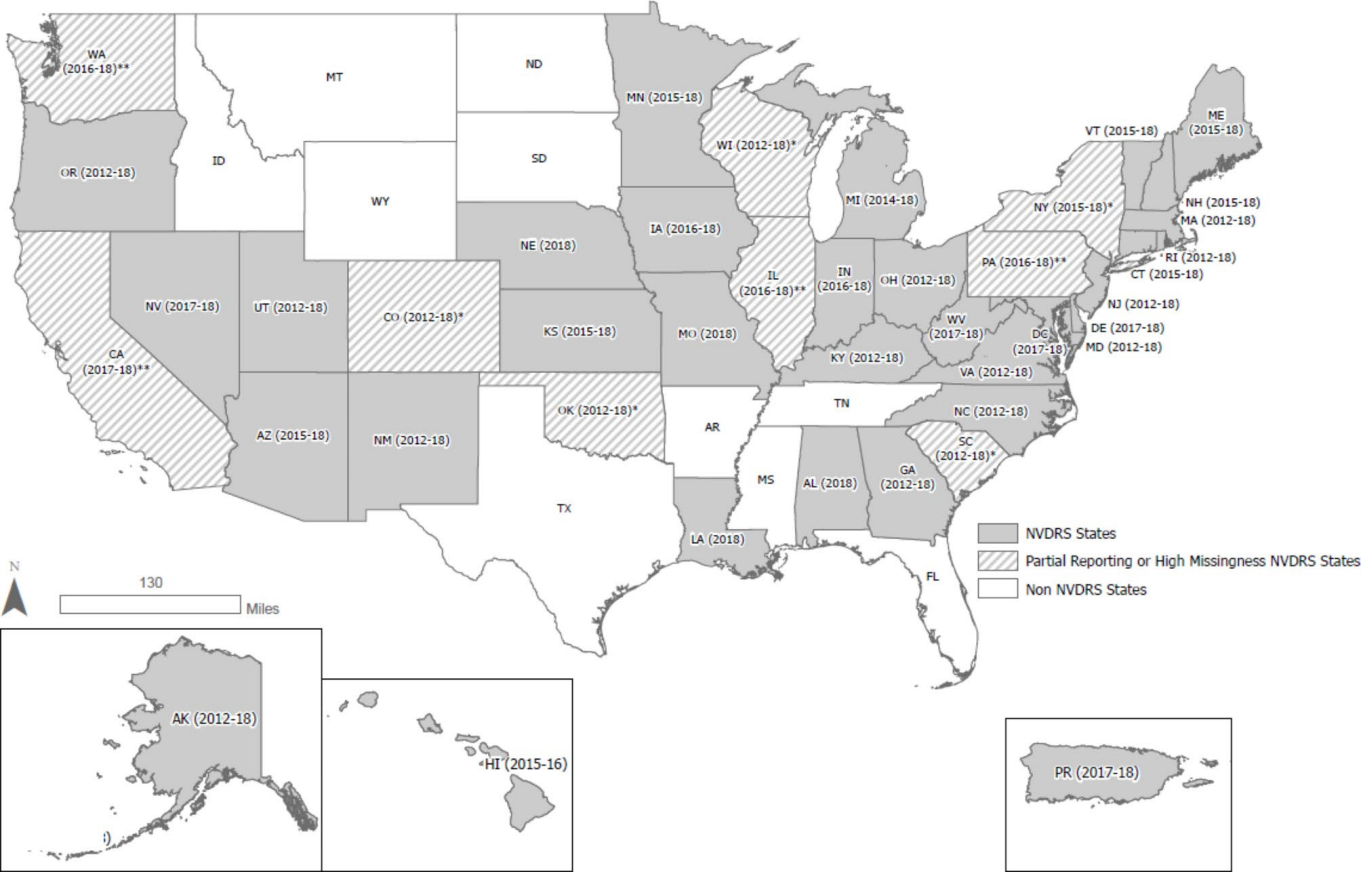
Map of Participating NVDRS States/Territories (2012–2018). NVDRS = National Violent Death Reporting System. *At least 10% of records in the state-year were missing 2 or more linkage variables (CO [2012–2018]; NY [2015–2018]; OK [2013, 2016–2018]; SC [2012]; WI [2012–2018]) **Partial reporting state; county level reporting (CA, 4 of 58 counties [2017], 21 counties (2018)]; at least 80% of violent death reporting (PA [2016–2018]; IL [2016–2018]; WA [2016–2017])

**NVDRS** provides comprehensive data regarding deaths by suicide, homicide, legal intervention, unintentional firearm injury, and deaths of undetermined intent that may be due to violence. Data consists of incident-linked information compiled from multiple sources, such as state death certificates, law enforcement reports, and coroner/medical examiner reports, into a national data repository coordinated by the CDC’s National Center for Injury Prevention and Control [21]. NVDRS began collecting data from seven states in 2003, incrementally adding states over the years. As of 2018, the final data year for this study, NVDRS collected data from 40 states, the District of Columbia and Puerto Rico [22].^1^ Of note, while most states contributed data on all qualifying deaths by 2018, some states were still participating as partial reporting states and thus did not provide complete data on all deaths. The current analysis includes NVDRS records for suicides and undetermined deaths among individuals 17 years of age and older, for all participating states in 2012–2018 (Fig. 1). While the focus of this study was on suicide, including deaths of undetermined intent allows for consideration of possible misclassification of suicides during data linkage. NVDRS does not include unique identifiers, however, the following minimally identifiable information, obtained primarily from death certificate records, is available for most states and years and was requested to conduct and/or assess the success of this linkage: first initial of last name; day of month of birth (DMB); age at death; date of death; state of death; sex; underlying cause of death (UCOD); multiple causes of death (MCOD); and military history indicator. NVDRS data for this study were accessed via the standard, publicly available Data Use Agreement (DUA) and process for accessing NVDRS-RAD files.

**VA-DoD MDR** is a joint effort between the VA and DoD to compile mortality data from the National Center for Health Statistics’ National Death Index (NDI) for all military and Veteran decedents [23]. NDI compiles state death certificate records and is the recognized national gold standard for ascertaining mortality status and cause of death in the United States [24]. A comprehensive search list of all service members and Veterans is compiled annually by the VA and DoD and provided to NDI to obtain all-cause mortality records. For this analysis, we obtained all VA-queried suicide or undetermined intent mortality records from MDR whose years and state of death coincided with the available NVDRS data from 2012 to 2018. For each death in MDR, Veteran status, first initial of last name, state of death, date of birth, date of death, sex, UCOD and MCOD were provided. To align with NVDRS variables, an MDR variable for day of month of birth and age at death were created, and the decedents’ date of birth was then removed from the linkage dataset, to avoid re-identifying NVDRS records, in accordance with the DUA. MDR data for this study were accessed via the standard DUA and process for accessing NDI records from the MDR, which is available to all VA and DoD principal investigators. The additional Veteran status indicator variable was obtained via a supplemental DUA with the VA Office of Suicide Prevention.

## Linkage process (Aim 1)

This multi-stage deterministic linkage [25] was limited to Veterans within MDR; NVDRS records were not limited based upon the NVDRS-documented military history variable, as we aimed to assess the accuracy of this variable. Given limited identifiable information available from NVDRS, multiple variables were used to identify matches between the datasets, commencing with strict criteria to identify exact matches and progressively relaxing criteria to increase yield. The *diyar* package was used in R version 4.0.5 [26, 27] to link variables in three separate stages. Records matched in one stage or substage were removed from both the NVDRS and MDR datasets ahead of the next stage. To operationalize this, we concatenated all variables included in each stage or substage to create a single linkage variable and stratified by both state and year, so only matches within each state-year strata (e.g., New York, 2015) were allowed.

**Fig. 2:**
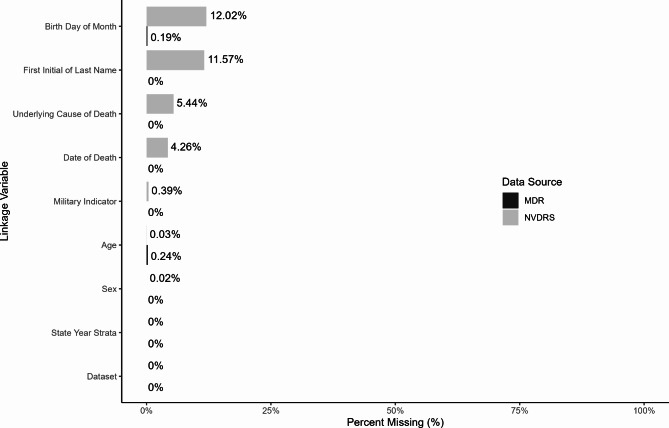
Missingness of Linkage Variables by Data Source. MDR = VA-DoD Mortality Data Repository; NVDRS = National Violent Death Reporting System. Day of month of birth and first initial of last name are not required variables, and thus are not reported by all states, which explains why missingness was notably higher for these two variables

Variables available in both datasets were first investigated for quality and completeness to inform the final set of linkage variables: age at death; sex; underlying cause of death; day of month of birth; first initial of last name; and date of death (Fig. 2). Additional variables were used to evaluate and confirm matches and to resolve duplicates: MCOD (up to 14 International Classification of Disease-10 codes); and the NVDRS military history indicator (yes, no, unknown).

**Fig. 3:**
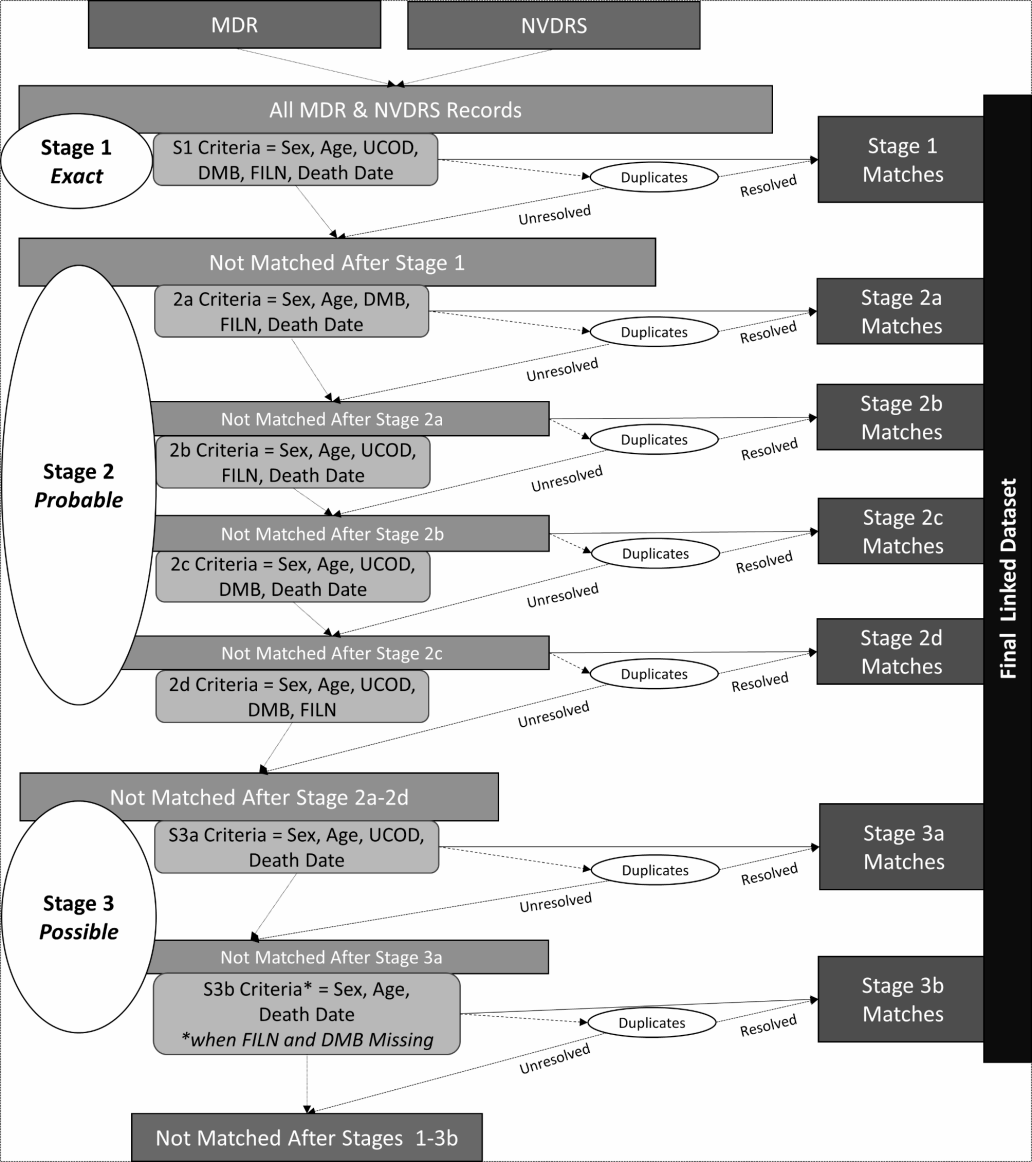
Deterministic Linkage Process Overview. MDR = VA-DoD Mortality Data Repository; NVDRS = National Violent Death Reporting System; UCOD = underlying cause of death; DMB = Day of Month of Birth; FILN = first initial of last name. Each state was stratified by year (i.e., state-year strata were used)

Linkage stages were conceptualized based on confidence of the record pair (i.e., a higher number of matching linkage variables conferred higher confidence in the match; Fig. 3). Stage 1, referred to as “exact matches,” had no discrepancies in matches for all linkage variables. Stage 2 “probable matches” comprised four sub-stages in which all but one of the linkage variables were included in match criteria: Stage 2a, excluded underlying cause of death from match criteria; next, Stage 2b excluded day of month of birth from match criteria; Stage 2c excluded first initial of last name; and finally, Stage 2d excluded date of death. Stage 3, referred to as “possible matches,” allowed 2 or more linkage variables to be discrepant. Specifically, in Stage 3a, both day of month of birth and first initial of last name were excluded as match criteria; in Stage 3b, only underlying cause of death was excluded from match criteria, but NVDRS records were limited to those where both day of month of birth and first initial of last name were missing. The variables removed iteratively within the second and third stages were selected due to high levels of missingness (4.3–12.0% of records) within some or all NVDRS state-year strata. Age and sex were not removed iteratively in stage 2 or 3 given low levels of missingness (<1% across all state-year strata) and our aim to assess accuracy of the NVDRS military history variable by age and sex.

## Evaluating linkage success (Aim 2a)

To evaluate the success of the linkage, we assessed duplicates, proportion of records linked, and linkage quality. Duplicates refer to instances where more than 2 records matched, such as 2 NVDRS records matching to the same MDR record or 1 NVDRS record matching to 2 MDR records. At each stage, duplicates were reviewed and reconciled manually by first comparing multiple cause of death (MCOD) fields between the MDR and NVDRS records. If a one-to-one match was not able to be identified by the presence of all MDR MCOD fields for a single NVDRS record, then additional variables were considered (i.e., linkage variables allowed to mismatch in Stages 2–3 and, subsequently, the NVDRS military indicator variable was considered). When the military indicator was considered, a duplicate was only resolved if only one NVDRS record indicated ‘yes’ for military history. When resolving duplicates, missing NVDRS fields were considered to be similar to present, matched fields; however, if all reviewed NVDRS fields were missing, the duplicate could not be resolved due to insufficient information. Records not matched and duplicate records unable to be resolved were pushed into the next stage of the linkage.

Next, we calculated the percentage of MDR records that linked to an NVDRS record. The percentage of linked records was calculated by dividing the total number of MDR records that were linked, by the total number available, overall and by stage and state-year. Additionally, as it was not feasible to link all records in partial reporting states, or those with high levels of missingness for key linkage variables (e.g., non-required variables: day of month of birth and first initial of last name), we also assessed the percentage of linked MDR records for ‘complete’ (i.e., complete reporting and low levels of missing data) state-years. Specifically, NVDRS state-years with > 10% of records missing two or more linkage variables were excluded, along with partial reporting state-years. Finally, a select set of key variables that were present in both data sources (i.e., UCOD, Death Date, MCOD) were compared for consistency and confirmation of linkage quality at select stages (i.e., 2a, 2d and 3b).

## Evaluating validity of NVDRS Military Variable (Aim 2b)

Finally, within complete state-years, we assessed the validity of the NVDRS military history variable by manner of death, by calculating its sensitivity, specificity, and accuracy, as compared to the MDR flag for Veteran status at the time of death. Validity was assessed overall, by sex, and by age group (17–39, 40–64, and 65+), separately for suicides and deaths of undetermined intent. Sensitivity was calculated by dividing the number of NVDRS Military = “Yes” records by the total number of Veteran MDR deaths in each group. Specificity was calculated by dividing the number of NVDRS records not linked to MDR by the total number of NVDRS Military = “No” records. Accuracy was calculated by dividing the total number of NVDRS records where the Veteran was correctly identified by the total number of NVDRS records. Positive and negative predictive value were also computed and measured the proportion of NVDRS records with a positive military history indicator who were Veterans and the proportion of NVDRS records without indication of military history who were not Veterans, respectively. Confidence intervals (CI; 95%) were computed for all estimates.

All analyses were conducted using R version 4.0.5. Maps were created using ArcGIS Pro [28]. This study was approved by the local VA Research and Development Committee and the local Institutional Review Board.

## Results

### Sample

Across MDR (*n* = 24,685) and NVDRS (*n* = 170,038) data sources, 194,723 suicide and undetermined death records were obtained from 40 US states, the District of Columbia, and Puerto Rico between 2012 and 2018 (Fig. 1). Most individuals died by suicide (92.31% of MDR; 87.05% of NVDRS). The suicide decedent sample was predominantly male (95.19% of MDR; 77.71% of NVDRS) and aged 40–64 years (41.66% of MDR; 45.81% of NVDRS); this was also true for the undetermined manner of death sample (Table 1).

**Table 1:** Characteristics of Eligible deaths, by Data source

	MDR		NVDRS	
	n	%	n	%
Manner of Death				
Suicide	22,787	92.31	148,020	87.05
Sex				
Male	21,692	95.19	115,025	77.71
Female	1,095	4.81	32,963	22.27
Missing	-	-	32	0.02
Military History				
Yes	22,787	100.00	24,770	16.73
No	-	-	115,781	78.22
Unknown or Missing	-	-	7,469	5.05
Age				
17–39	4,223	18.53	54,011	36.49
40–64	9,494	41.66	67,803	45.81
65+	9,012	39.55	26,157	17.67
Missing or Invalid	58	0.25	49	0.03
Manner of Death				
Undetermined	1,898	7.69	22,018	12.95
Sex				
Male	1,726	90.94	14,267	64.80
Female	172	9.06	7,751	35.20
Military History				
Yes	1,898	100.00	1,750	7.95
No	-	-	19,098	86.74
Unknown or Missing	-	-	1,170	5.31
Age				
17–39	395	20.81	8,803	39.98
40–64	1,053	55.48	11,420	51.87
65+	448	23.60	1,795	8.15
Missing or Invalid	2	0.11	-	-
Total	24,685	100	170,038	100

Note. MDR = Mortality Data Repository; NVDRS = National Violent Death Reporting System

Age, sex and manner of death for each data source were determined from that data source. Manner of death for MDR is determined based on underlying cause of death, whereas manner of death for NVDRS is determined by the manner of death variable. Military history in NVDRS is determined from the military history variable, whereas all MDR records in this analysis were Veterans

## Data quality

Higher levels of missingness for key linkage variables were observed in NVDRS (up to 12.02%) compared to MDR (up to 0.24%; Fig. 2) data. For NVDRS, day of month of birth and first initial of last name were most frequently missing; this was expected as these are not required reporting elements. Underlying cause of death and date of death were also missing for 5.44% (*n* = 9,246) and 4.26% (*n* = 7,237) of NVDRS records, respectively, but were not missing at all in MDR. These results partially informed the selection of criteria to relax in each stage of the linkage.

## Linkage success

Duplicates occurred infrequently across stages; overall, there were 23 total duplicate groups of 2 or more records reviewed across stages, comprising 76 individual records (*n* = 28 MDR; *n* = 48 NVDRS). Most duplicates were resolved based on MCOD review (*n* = 15 pairs; 65.22% of resolutions); 21.74% of duplicates were resolved with the military history indicator (*n* = 5 pairs in Stages 2d and 3), and 13.04% were resolved based upon review of date of death (*n* = 3 pairs in Stage 2d).

**Fig. 4:**
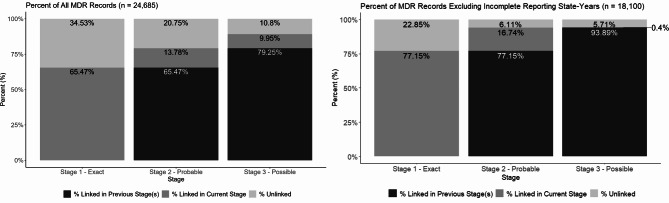
Percent of MDR Records Linked and Unlinked, by Stage

**Fig. 5:**
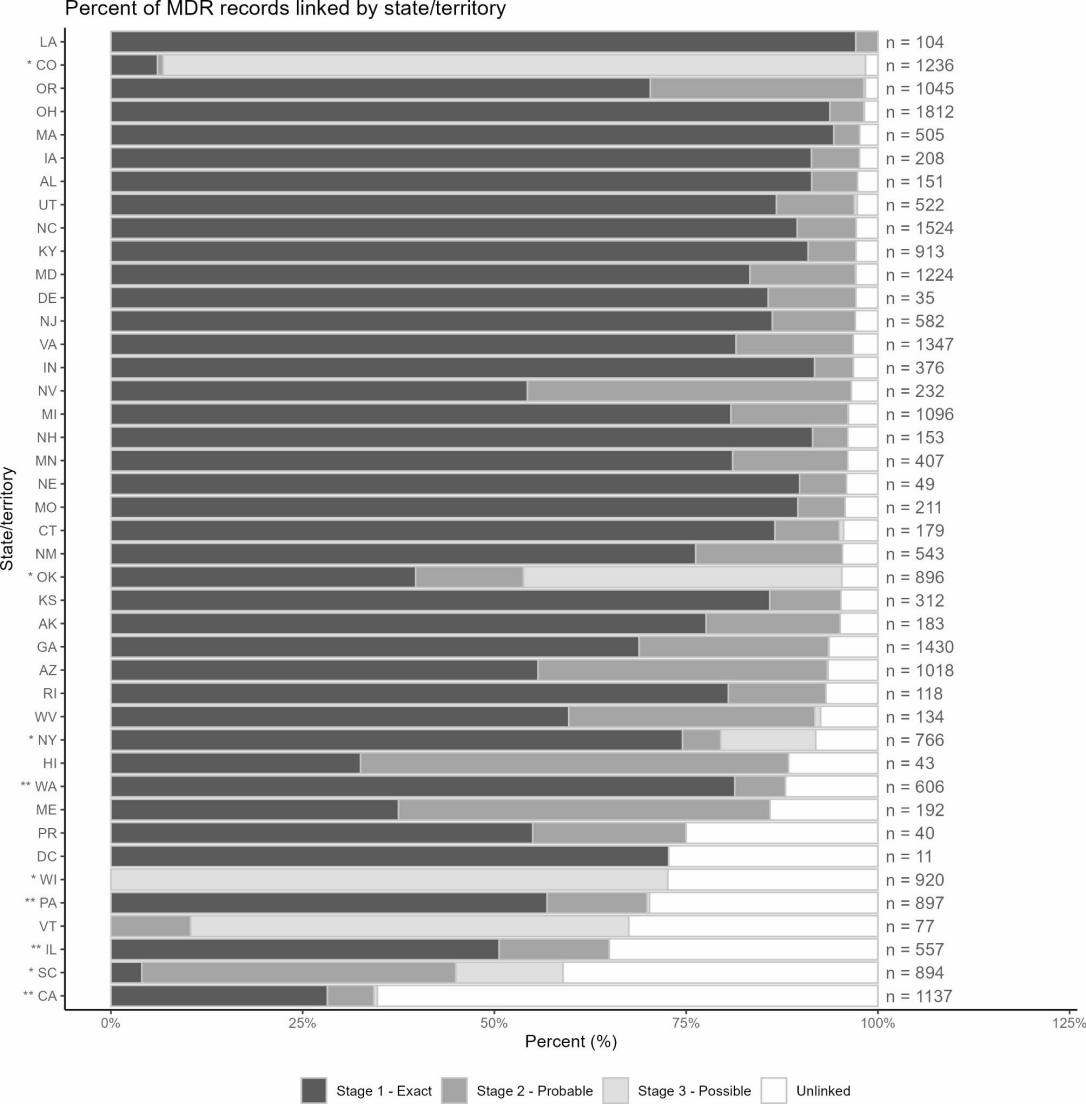
Percent of Records Linked , by Stage and State/Territory. *High missingness state in which at least 10% of records in the state-year were missing 2 or more linkage variables (CO [2012–2018]; NY [2015–2018]; OK [2013, 2016–2018]; SC [2012]; WI [2012–2018]). **Partial reporting state: county level reporting (CA, 4 of 58 counties [2017], 21 counties (2018)]; minimum reporting requirement of at least 80% of violent deaths (PA [2016–2018]; IL [2016–2018]; WA [2016–2017])

Across all 189 state-year strata, 22,019 (89.20% of MDR records) matches were identified (65.47% exact, 13.78% probable, 9.95% possible; Fig. 4a). The percent of records linked ranged from 34.74 to 100% across states ([mean = 89.55%, standard deviation = 13.68%], Fig. 5). There were 4 states (10 state-year strata) in our dataset with partial reporting requirements and 5 states (23 state-year strata) with high missingness (Fig. 1). When excluding these state-years, the overall percentage of MDR records matched increased to 94.29% (*n* = 17,067; 77.15% exact, 16.74% probable, 0.40% possible; Fig. 4b).

When UCOD was allowed to mismatch, the majority of linked pairs matched because UCOD was missing from NVDRS (53.98% of 1,293 pairs in Stage 2a and 72.89% of 284 pairs in Stage 3b). Likewise, of the pairs matched in Stage 2d (*n* = 843), when death date was allowed to mismatch, the majority (89.44%) matched due to missing NVDRS dates. When NVDRS dates were present, most MDR and NVDRS death date differences were within 30 days (*n* = 70 pairs; 78.65%); only 19 pairs had death date differences of more than 30 days.

### Validity of NVDRS military history variable

Overall accuracy of the NVDRS military indicator variable was 87.29% for suicides and 90.74% for deaths of undetermined intent (Table 2). Accuracy varied moderately across demographic groups and manner of death (range: 79.77 − 93.67%) and was highest among undetermined intent deaths as compared to suicides (90.74% vs. 87.29%) and among females (91.38% for suicides, 93.43% for undetermined intent), and those 17–39 years of age (89.20% for suicides, 93.67% for undetermined intent). However, sensitivity was higher for males than for females, as was positive predictive value for both suicides and undermined intent deaths. Conversely, specificity and negative predictive value were higher for females across both manners of death, driving the higher overall accuracy in this group. While sensitivity increased with increasing age, irrespective of manner of death, specificity was notably lower among the oldest group and similar among those 17–39 and 40–64. Resultingly, positive predictive value was highest in the age 40–64 years group and was lowest for the youngest group whereas negative predictive value was similar across all age groups, especially for suicide decedents.

**Table 2:** Validity of NVDRS Military History Variable for Suicide and undetermined intent deaths, overall and stratified by sex and age at death

	Accuracy (95% CI)	Sensitivity (95% CI)	Specificity (95% CI)	Positive Predictive Value (95% CI)	Negative Predictive Value (95% CI)	
Suicide Deaths						
** Overall**	87.29% (87.08%, 87.49%)	85.01% (84.44%, 85.57%)	87.66% (87.45%, 87.88%)	70.94% (70.28%, 71.59%)	97.87% (97.76%, 97.97%)	
** By Sex**						
Female	91.38% (91.02%, 91.73%)	62.06% (58.47%, 65.56%)	92.29% (91.94%, 92.63%)	63.77% (60.15%, 67.28%)	98.86% (98.71%, 99.00%)	
Male	86.15% (85.91%, 86.38%)	86.15% (85.58%, 86.70%)	86.15% (85.88%, 86.40%)	71.23% (70.56%, 71.89%)	97.52% (97.39%, 97.64%)	
** By Age**						
17–39	89.20% (88.89%, 89.51%)	77.58% (76.20%, 79.09%)	90.11% (89.80%, 90.42%)	58.64% (57.07%, 60.20%)	98.33% (98.19%, 98.47%)	
40–64	88.78% (88.50%, 89.06%)	81.10% (80.13%,82.03%)	89.94% (89.65%, 90.23%)	81.67% (80.71%, 82.60%)	97.55% (97.39%, 97.70%)	
65+	79.77% (79.20%, 80.34%)	92.77% (92.09%, 93.41%)	73.93% (73.18%, 74.66%)	68.16% (67.15%, 69.17%)	97.56% (97.24%, 97.86%)	
**Undetermined Intent Deaths**						
** Overall**	90.74% (90.31%, 91.16%)	79.91% (77.63%, 82.05%)	91.55% (91.13%, 91.97%)	69.92% (67.52%, 72.24%)	98.59% (98.39%, 98.77%)	
** By Sex**						
Female	93.43% (92.80%, 94.03%)	53.40% (43.30%, 63.30%)	94.07% (93.46%, 94.64%)	52.88% (42.84%, 62.46%)	99.28% (99.03%, 99.48%)	
Male	89.30% (88.74%, 89.85%)	82.17% (79.89%, 84.30%)	90.08% (89.51%, 90.64%)	71.19% (68.73%, 73.57%)	98.17% (97.88%, 98.43%)	
** By Age**						
17–39	93.67% (93.09%, 94.21%)	76.77% (71.54%, 81.46%)	94.36% (93.80%, 94.88%)	70.59% (65.29%, 75.51%)	99.08% (98.82%, 99.29%)	
40–64	89.53% (88.90%, 90.13%)	77.95% (74.83%, 80.85%)	90.50% (89.88%, 91.11%)	71.31% (68.10%, 74.37%)	98.24% (97.93%, 98.51%)	
65+	83.33% (81.26%, 85.27%)	89.60% (85.13%, 93.10%)	81.95% (79.57%, 84.15%)	65.88% (60.57%, 70.92%)	98.09% (97.00%, 98.87%)	
Note. NVDRS = National Violent Death Reporting System; CI = Confidence Interval						

These analyses exclude deaths from the *n* = 33 state-years with incomplete reporting or high missingness and 41 decedents with missing age when computing estimates by age group

## Discussion

We report successful, reproducible methods to link NVDRS and VA data while maintaining anonymity of NVDRS records. This achieves a long-desired goal to enhance Veteran suicide surveillance [19]. The multi-stage, deterministic linkage approach used to link 2012–2018 VA-DoD MDR and NVDRS suicide and undetermined intent death records resulted in an 89.20% match rate overall, with even greater success observed in states with complete NVDRS data (94.29% match rate). For context, the biennial SEER-Medicare linkage, which uses personal identifiable information, achieves a linkage rate of approximately 95% [29]. Our comparable success without the use of personal identifiers is likely due to the availability of key linkage variables and the low levels of linkage variable missingness and error across data sources. Deterministic approaches are recognized to be both effective and efficient in such “information rich” scenarios [25, 30]. Since 2018, NVDRS has expanded to all 50 states, the District of Columbia, and Puerto Rico, although some states are still working towards full, statewide coverage. Furthermore, some states previously participating as partial reporting states (i.e., Illinois, Pennsylvania, Washington) now report all violent deaths from their states. With such improvements, our findings suggest that linkage of more recent data will likely be even more successful; validation of this approach is indeed feasible and therefore warranted using mortality data from 2019 to 2022 (most current data available). Moreover, there are additional opportunities to further improve linkage by using additional VA data. For example, although missing age in MDR was not imputed prior to linkage, future linkages could do so by adding information from alternate data sources (e.g., VA Profile) [31].

Despite the overall success of the linkage and anticipated improvements over time, we identified states for which linkage was more challenging. Specifically, some states (e.g., Colorado, Oklahoma) had high levels of overall linkage, but a greater proportion of probable or possible matches. Such states are likely to have higher missingness (i.e., at least 10% of records in the state-year missing 2 or more linkage variables), which in some cases may be due to those states opting not to report day of month of birth or first initial of last name, as doing so is not required. While our review of linked records suggests that even ‘possible’ matches are highly accurate, such states could benefit from considering the utility of reporting such variables in the future, especially if they have a large Veteran population. Conversely, states for which we observed lower overall linkage, irrespective of linkage stage, were largely partial reporting states (e.g., Pennsylvania); we anticipate linkage rates in those states will increase substantially as they shift to full coverage.

The opportunity to evaluate the NVDRS military history variable in this analysis was also novel and informative. The accuracy of the military history variable in NVDRS and its utility for studying Veteran suicide has been a noted concern for researchers. Specifically, active-duty death records are included in NVDRS, and military service history may be misclassified on death certificate records, especially among certain groups (e.g., low sensitivity for female Veterans) [32]. While some prior studies have concluded that this expected misclassification of Veteran status is likely to have limited impact on suicide rates estimated from NVDRS data [33], there could be greater impact for understanding differential suicide risk factors between Veterans and non-Veterans, especially among groups where misclassification is higher. Indeed, our findings confirm that, while overall sensitivity and specificity were acceptable (85.01% and 87.66%, respectively, for suicides), sensitivity was not optimal for female (62.06%) and younger (age 17–39, 77.58%) Veterans who died by suicide. Given the likelihood for differences between Veterans in these groups who are and are not identified within NVDRS (e.g., length service and social connection may impact reporting of military history on death certificate), there is potential for analyses conducted with these groups to be biased. With increasing concerns regarding rising suicide risk among both of these Veteran subgroups [1], this linkage approach provides an important opportunity to correct such bias and conduct novel analyses to inform targeted prevention efforts.

### Limitations

This study does have limitations. First, we consider limitations inherent to the data sources used (i.e., NVDRS and MDR). With regard to data access, the MDR is restricted to VA and DoD-affiliated principal investigators. However, there is no restriction to investigators partnering with academic institutions and/or other organizations on such research. With regard to accuracy and completeness, there are two considerations to note. First, the MDR is subject to the same limitations inherent to all NDI matches; NDI does reject some search records and all matched records are returned with match probability scores requiring the VA/DoD to implement an algorithm to identify what they believe to be true matches [34]. Second, while the VA and DoD compiles the annual NDI search list using multiple data sources, it is recognized that there are limitations to capturing older Veterans (i.e., those over 67 years in 2018) due to incomplete electronic personnel data [34]. It is thus possible that some deceased Veterans, especially older Veterans, will not be captured in the MDR due to imperfect matching results and thus cannot be linked to NVDRS records. This may partially explain the lower specificity observed for the NVDRS military history variable among older Veterans. Finally, both data sources have a lag in availability that limits the timeliness of mortality surveillance efforts; while this is common and universal to all who use these data sources for such efforts it is important to consider the public health impact of such delays. Specifically, NVDRS data is typically delayed by 19 months and MDR data by 2 to 3 years. There are some additional limitations to consider with regard to this data linkage. Specifically, it is possible that some matches identified were false positives, though review of linked records indicates this is likely uncommon. Moreover, as discussed above, linkage success is dependent upon state-level data quality and completeness, which can change over time.

Despite these considerations, these findings suggest that recurring linkage of VA, DoD, and NVDRS data is feasible and could enhance surveillance of Veteran suicide—and thus bolster prevention as well—in line with the National Strategy for Preventing Veteran Suicide [3] and the 2024 National Strategy for Suicide Prevention [20]. The resulting database can be used to facilitate a wealth of informative analyses to accurately identify Veterans who died by suicide, overall and by history of VHA use; to expand knowledge on the accuracy of behavioral health variables in NVDRS at a national level [18]; and to examine circumstances preceding death to inform enhanced and targeted suicide prevention efforts in clinical and community settings. Moreover, while our focus was on suicide, the methods presented here could be expanded to include additional causes of violent death (e.g., homicides, unintentional firearm injuries), to further enhance violent death surveillance research in the Veteran population—with the ultimate goal of saving lives.

## Conclusions

The linkage of these rich data sources offers a valuable resource to inform Veteran suicide prevention efforts. While there has been considerable investment into strengthening data sources on Veteran suicide, each source has specific strengths and omissions that have sometimes precluded researchers from ascertaining a comprehensive understanding of risk factors and characteristics surrounding each death. This large-scale linkage study shows that it is feasible to merge information across the VHA and NVDRS systems by incident. With this new resource, researchers can examine details spanning the decedent’s health, relationships, life-stress circumstances, mental health service use, mechanism of death, and disclosures. We anticipate that the methods established in this study have the potential to support novel epidemiologic studies on Veteran suicide deaths that can inform prevention efforts.

## Data Availability

The datasets generated during the current study cannot be made publicly available due to requirements within the DUA in place between the CDC and the investigator team. Rather, this manuscript outlines a procedure for which other investigators could create a similar dataset upon receiving appropriate DUAs from the CDC (i.e., for NVDRS-RAD data) and the VA/DoD (i.e., for MDR data).

## Abbreviations

CDC: Centers for Disease Control and Prevention
CI: Confidence Interval
DMB: Day of Month of Birth
DoD: Department of Defense
MCOD: Multiple Cause of Death
MDR: Mortality Data Repository
NVDRS: National Violent Death Reporting System
RAD: Restricted Access Database
SDOH: Social Determinant of Health
UCOD: Underlying Cause of Death
VA: Department of Veterans Affairs
VHA: Veterans Health Administration

## Footnotes

1 Since 2018, NVDRS has expanded to full implementation of at least partial reporting across all 50 states, the District of Columbia, and Puerto Rico.
